# Stride-to-Stride Variability of the Center of Mass in Male Trained Runners After an Exhaustive Run: A Three Dimensional Movement Variability Analysis With a Subject-Specific Anthropometric Model

**DOI:** 10.3389/fspor.2021.665500

**Published:** 2021-05-24

**Authors:** Felix Möhler, Bernd Stetter, Hermann Müller, Thorsten Stein

**Affiliations:** ^1^BioMotion Center, Institute of Sports and Sports Science (IfSS), Karlsruhe Institute of Technology, Karlsruhe, Germany; ^2^Sports Orthopaedics, Institute of Sports and Sports Science (IfSS), Karlsruhe Institute of Technology, Karlsruhe, Germany; ^3^Training Science, Department of Sports Science, Justus-Liebig-Universität Giessen, Giessen, Germany

**Keywords:** motor control, tolerance noise covariation (TNC), uncontrolled manifold (UCM), locomotion, mid-distance running

## Abstract

The motion of the human body can be described by the motion of its center of mass (CoM). Since the trajectory of the CoM is a crucial variable during running, one can assume that trained runners would try to keep their CoM trajectory constant from stride to stride. However, when exposed to fatigue, runners might have to adapt certain biomechanical parameters. The Uncontrolled Manifold approach (UCM) and the Tolerance, Noise, and Covariation (TNC) approach are used to analyze changes in movement variability while considering the overall task of keeping a certain task relevant variable constant. The purpose of this study was to investigate if and how runners adjust their CoM trajectory during a run to fatigue at a constant speed on a treadmill and how fatigue affects the variability of the CoM trajectory. Additionally, the results obtained with the TNC approach were compared to the results obtained with the UCM analysis in an earlier study on the same dataset. Therefore, two TNC analyses were conducted to assess effects of fatigue on the CoM trajectory from two viewpoints: one analyzing the CoM with respect to a lab coordinate system (PV_lab_) and another one analyzing the CoM with respect to the right foot (PV_foot_). Full body kinematics of 13 healthy young athletes were captured in a rested and in a fatigued state and an anthropometric model was used to calculate the CoM based on the joint angles. Variability was quantified by the coefficient of variation of the length of the position vector of the CoM and by the components Tolerance, Noise, and Covariation which were analyzed both in 3D and the projections in the vertical, anterior-posterior and medio-lateral coordinate axes. Concerning PV_lab_ we found that runners increased their stride-to-stride variability in medio-lateral direction (1%). Concerning PV_foot_ we found that runners lowered their CoM (4 mm) and increased their stride-to-stride variability in the absorption phase in both 3D and in the vertical direction. Although we identified statistically relevant differences between the two running states, we have to point out that the effects were small (CV ≤ 1%) and must be interpreted cautiously.

## Introduction

One of the key questions in the field of motor control is how humans are able to perform skilled movements. Competitive sports might be seen as performing movements in perfection: a gymnast, for example, is able to perform complex movements with maximal aesthetics, and an endurance athlete performs his/her movements with maximal efficiency. With respect to that, variability might be seen as counterproductive, since it causes deviations from the singular “optimal movement” in a given situation. However, a certain amount of variability is desirable since it could avoid overload injuries (Hamill et al., 2012). So, the benefit of variability might depend on the variable we are looking at. It was shown that parameters such as movement speed, footwear, expertise, and fatigue affect movement variability (Jordan and Newell, 2008; Fuller et al., 2016; García-Pinillos et al., 2020). Since fatigue is an unavoidable phenomenon in endurance sports, the question arises as to how fatigue affects motor variability and whether athletes are still able to perform their movements with the same consistency in a fatigued state.

Variability analyses are well-established within the field of motor control, with different degrees of complexity (Sternad, 2018). Namely, these are the Goal Equivalent Manifold (GEM, Cusumano and Cesari, 2006), the Uncontrolled Manifold (UCM, Scholz and Schöner, 1999) and the Tolerance, Noise and Covariation (TNC, Müller and Sternad, 2004) approaches, all of which allow analysis of functional structure and repartition of movement variability. Common to these approaches is the examination of a task-relevant performance variable (PV). Its value should show low variability and stay close to the optimum over several movement repetitions to ensure successful task completion. The execution of the movement is described by execution variables (EV's). A main difference between the TNC approach and the UCM and GEM approach is the fact that the TNC analyses the variability on the level of the PV whereas the UCM and GEM analyze the variability on the level of EV. There exist different kinds of variability: If variability among the EV's does not increase variability of the PV it is supposed to be “good,” since this variability could be essential for adaptations or motor learning (Latash et al., 2010). On the other hand, variability among the EV's which affects the PV is considered to be “bad” since it causes deviations from the desired PV-value. To analyze the effect of the variability of certain EV's, the PV has to be formulated as a function of the EV's. One example of a PV might be hitting a target, e.g., a specific field on a dartboard, with a dart. This PV could be described as a function of the EV's release angle and velocity (Müller and Sternad, 2004).

The approaches mentioned above have mostly been applied to movements with a restricted number of degrees of freedom and far less often to whole-body movements. Some recent studies have analyzed walking using diverse analyses (GEM: Dingwell, Bohnsack-McLagan, and Cusumano 2018; UCM: Yamagata et al., 2019; and TNC: Hamacher et al., 2019). Using a GEM approach, Dingwell et al. (2018) showed that the structure of stride-to-stride variability was speed-dependent: variability affecting the PV decreased with speed. Yamagata et al. (2019) showed a relationship between incident falls and stride-to-stride-variability in older adults using an UCM approach. The study by Hamacher et al. (2019) investigated the stride-to-stride variability in walking by means of the TNC approach and has highlighted the usefulness of this approach for gaining deeper insight into related motor adaptations. Using the TNC approach, Hamacher et al. (2019) found decreases in gait variability during dual task walking due to the component “noise.” However, there are only a few studies analyzing the stride-to-stride variability in running. Dingwell et al. (2018) found tighter control in running compared to walking as indicted by quicker corrections. In our earlier studies we found higher stride-to-stride variability in novices compared to experts and only slight changes due to fatigue using an UCM approach (Möhler et al., 2019, 2020). Brahms et al. (2020) analyzed the coefficient of variation (CV) of several spatiotemporal parameters (stride time and length, contact time) and peak acceleration during an overground run with constant speed. They found no effects of fatigue, which is interpreted as a confirmation for the insensitivity of linear variability measures. Skowronek et al. (2021) investigated the effects of an aerobic running protocol on jump rhythm using the Optojump Next system. They found that the rhythm of movement is impaired by the anaerobic fatiguing protocol. To date, the TNC approach has not been used to study running.

As stated above, a PV has to be determined first in all mentioned approaches and should be kept constant between movement repetitions. In the case of endurance running, it can be assumed that runners adopted a subject-specific optimal running style over years of training (Williams and Cavanagh, 1987; Moore, 2016). This optimal running style should be kept constant from stride-to-stride if the ambient conditions do not change. The CoM trajectory can be used to describe this running style (Blickhan, 1989; Dutto and Smith, 2002) and is one of several biomechanical parameters which influence running economy (Williams and Cavanagh, 1987; Tartaruga et al., 2012; Moore, 2016). It has been shown that during a run to fatigue with self-selected speed, runners adjust their speed rather than their vertical CoM position, which underlines the importance of keeping this parameter constant (Girard et al., 2013). When running on a treadmill however, speed is mostly fixed and runners are thus not able to adjust their running speed. Consequently, the question is whether and how runners adjust their CoM trajectory when they are not able to adjust their speed when they become fatigued and how fatigue affects the variability of the CoM trajectory.

The CoM trajectory can be described with respect to different reference points when running on a treadmill. The origin of the lab coordinate system as a fixed reference point (Möhler et al., 2019) is one possible viewpoint. However, Moore et al. (2016) found that the alignment of the ground reaction force with the leg axis led to increases in running economy. This seems plausible, since the runner tries to accelerate his/her body (represented by the CoM) forwards and upwards against gravity by pushing his/her body over the legs (Heise and Martin, 2001), so the description of the CoM trajectory in a body-related coordinate system (e.g., relative to the pushing foot) might be better suited as a relevant PV during running than the CoM trajectory in a lab coordinate system (e.g., relative to an arbitrarily chosen point in the lab). Besides, even if a 3D analysis is desirable (Papi et al., 2015), the separate analysis of the three dimensions as complementary measures could provide valuable information since the observed variability could be repartitioned in the three directions. However, this is not easy to implement with an analysis in the execution space (as with the UCM analysis) since the EV's must have the same units (e.g., joint angles in degrees vs. foot position in meters) and a new model has to be built up for each direction (Latash et al., 2007; Müller and Sternad, 2009). In contrast to the UCM approach, the TNC approach allows for the combination of EV's with different units, since the analysis is performed in the result space (Müller and Sternad, 2009). Whereas UCM analysis is applicable to a single data set, TNC analysis can only reveal changes in movement variability between two states (Müller and Sternad, 2003, 2004). However, this is suitable for looking at differences between a fatigued and a non-fatigued state.

In this study, data from Möhler et al. (2019) were re-analyzed using the TNC approach to gain a deeper insight into changes in motor coordination due to running induced fatigue. Effects of fatigue on running mechanics were shown to be dependent on the type of fatigue, as Fischer et al. (2015) found clear effects of a high intensity short-time fatigue protocol on spatiotemporal parameters and Vernillo et al. (2016) found no effects of an extreme ultra-marathon on the spatiotemporal parameters observed. In our study, we analyze the effects of an anaerobic run to exhaustion. The purpose was to investigate if and how runners adjust their CoM trajectory due to high intensity anaerobic fatigue (~4 min at ~19 km/h) and how this fatigue affects the variability of the CoM trajectory. Additionally, we wanted to compare our results to the ones obtained with the UCM approach in our earlier study on the same dataset (Möhler et al., 2019). Therefore, we calculated the TNC approach for two PV's: PV_lab_ as the CoM with respect to a fixed point in order to compare our results to the ones obtained with the UCM and PV_foot_ as the CoM with respect to the right foot in order to choose a PV which potentially better suited to functionally study running. So, we obtained two vectors which were described in dependence of the joint angles as EV. The CV of the length of these vectors was observed as a measure of variability of the CoM trajectory. Our hypotheses for the two PV were: (1) According to our previous study (Möhler et al., 2019), the TNC analysis would reveal no effects of fatigue when looking at PV_lab_ in 3D. (2) Based on previous biomechanical studies which found effects of fatigue on different joint angles (Winter et al., 2017) as well as increases in variability with fatigue on spatio-temporal parameters and their variability (García-Pinillos et al., 2020) the TNC analysis looking at PV_foot_ would show changes in CoM trajectory as well as increases in variability with fatigue.

## Methods

### Used Dataset

A description of the study design is given in the following section. Further details can be found in Möhler et al. (2019). The sample consisted of 13 healthy young experienced male runners (age: 23.5 ± 3.6 years, BMI: 20.6 ± 1.7 kg/m^2^, 7.2 ± 3.2 years of running training, 10 km record 32:59 ± 01:19 min). Inclusion criteria were an active membership in a running club for at least 2 years, a 10 km record below 35 min, a minimum training volume of 50 km/week during the 8 weeks before the measurements. Exclusion criteria were recent injuries or pain in the lower limbs. A total of 22 anthropometric measures were taken manually from each participant and 41 reflective markers were attached to anatomical landmarks to perform an inverse kinematics calculation using the Alaska Dynamicus full body model (Härtel and Hermsdorf, 2006). One week prior to the biomechanical measurement, participants came to the lab to perform a lactate threshold test. Following the critical power concept (Monod and Scherrer, 1965), their individual fatigue-speed was determined. This speed was at 19.27 ± 0.72 km/h. On the day of the measurement, participants performed a standardized treadmill familiarization [6 min of walking (Matsas et al., 2000), 6 min of running (Lavcanska et al., 2005)]. Afterwards, participants ran on the treadmill at their individual fatigue speed until voluntary exhaustion. Participants reached voluntary exhaustion at this speed after 4:06 ± 0:52 min. Their perceived fatigue was reported as 19.6 ± 0.65 on the Borg Scale (Borg, 1982). For each participant, a minimum of 20 consecutive step cycles were collected at the beginning, 20 seconds after the fatigue speed was reached (PRE state) and end of the run, when the participant indicated exhaustion (POST state). Due to data issues, only 19 consecutive gait cycles per participant could be analyzed. Based on marker data (heel and toe marker, Leitch et al., 2011), the right stance phase was determined. Since the running mechanics could change with the foot strike pattern (Lieberman et al., 2010) we verified that foot strike patterns did not change from PRE to POST (angle between longitudinal foot axis and ground PRE: 3.16°, POST: 3.76°, *p* = 0.164). Data were cut to the right stance phase and time-normalized to 100 time points using a cubic spline interpolation. The time-normalized stance phase was then further divided into absorption phase (1–50%) and propulsion phase (51–100%) (Cavanagh and Lafortune, 1980; da Rosa et al., 2019). These data serve as input for the following TNC analysis.

### TNC Analysis

In order to perform a TNC analysis, one has to define EV and a PV and a forward model linking the EV with the PV. The joint angles were defined as EV. The PV is supposed to be a variable which is controlled in a way that its value remains constant over several trials (in our case: strides). The steps and choices necessary to perform a TNC analysis are described in the following sections. We first describe our EV, PV_lab_, and PV_foot_, then our anthropometric model and finally the decomposition of variability in T, N, and C.

#### PV_Lab_

**Figure 1:**
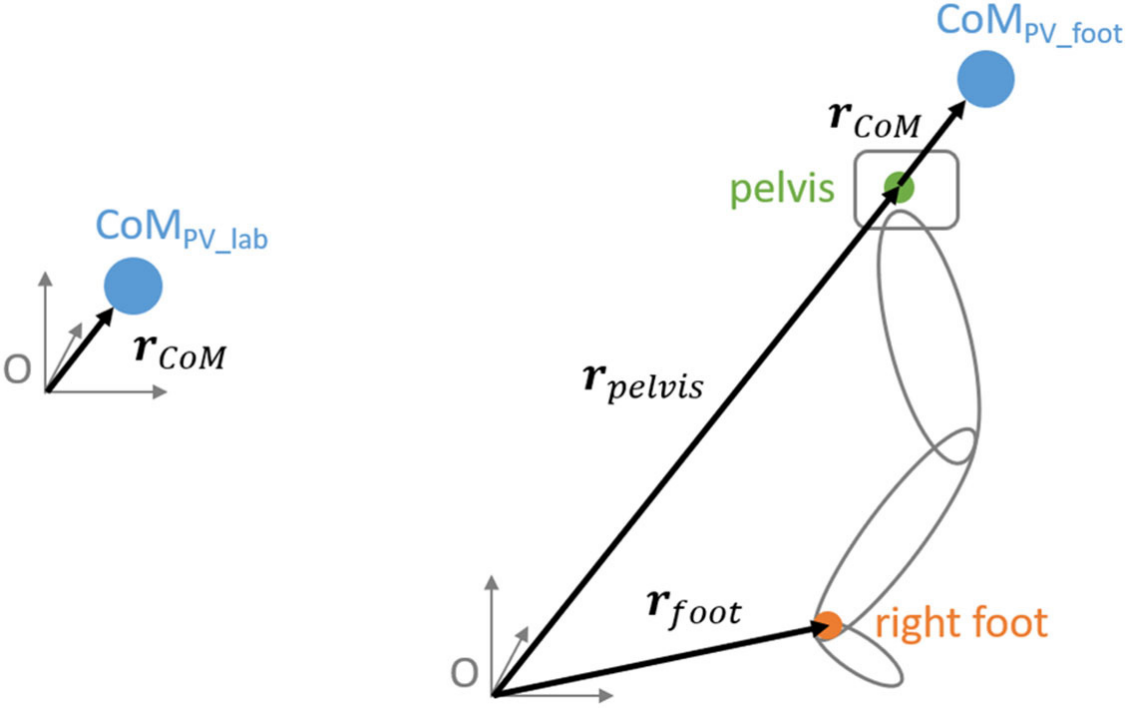
Drawing illustrating the calculation of the two PV's [see equations (2) and (3)]. To the left side, the definition of PV_lab_ as the CoM relative to the origin. To the right side the definition of PV_foot_. The right leg is shown in gray with the midpoint of the malleolus markers (right foot) in orange and the midpoint of the pelvis in green.

In accordance with our previous UCM analysis (Möhler et al., 2019), we defined the CoM trajectory relative to a fixed point as PV_lab_ (**r**PV_lab_), respectively, the length of the vector (euclidean norm) pointing from this fixed point to it [see Figure 1 and Equation (2)]. The chosen coordinate axes were classified as: pointing parallel to the treadmill belt (x-direction, anterior-posterior), vertical (z-direction), or perpendicular to these two axes (y-direction, medio-lateral). Therefore, x and z represent physically meaningful directions (running direction, gravity).

The coefficient of variation (CV) was calculated as a measure for stride-to-stride variability. Thus, the degree to which the PV is kept constant over the 19 cycles could be quantified. CVs were calculated for the 3D length of the vector and on the projections in the individual directions (anterior-posterior, medio-lateral and vertical direction).

#### PV_Foot_

We defined the CoM trajectory relative to the right foot as PV_foot_ (***r***PV_foot_), more precisely the length of the vector pointing from the right foot to the CoM [see Figure 1 and Equation (3)]. The chosen coordinate axes were classified as: pointing parallel to the treadmill belt (x-direction, anterior-posterior), vertical (*z*-direction) or perpendicular to these two axes (y-direction, medio-lateral). The length of the vector pointing from the right foot to the CoM trajectory was then calculated (euclidean norm).

CV's of PV_foot_ were calculated in the same manner as for PV_lab_.

#### 3D Anthropometric Model

To perform a TNC analysis, a forward model is required which links the PV (CoM trajectory) with the EV's (joint angles). We used the same subject-specific 3D forward model as used for our previous UCM analysis (Möhler et al., 2019) consisting of 17 segments and 50 degrees of freedom (47 segmental angles and 3 hip rotations). The 50 degrees of freedom of the anthropometric model were defined as EV's. The model is based on the Hanavan model (Hanavan, 1964) and was modified by including a neck and a hip segment. The shapes of the segments were defined using 36 subject-specific anthropometric measurements, thereof 21 measured manually and 15 determined through the marker data. By assuming a constant density distribution (Ackland et al., 1988), the segment's masses could be determined *via* volume integration. The whole-body CoM (***r***_*CoM*_, see Figure 1) was calculated as a weighted sum:

(1) $$\begin{array}{r} r_{CoM}=\;\frac{1}{\overset{N}{\underset{i=1}{\sum }}V_{i}}*\overset{N}{\underset{i=1}{\sum }}r_{i}V_{i} \end{array}$$

With *N* as the number of segments; *V*_*i*_ as the volume of segment *i*; ***r***_*i*_ as the vector of the center of gravity of segment *i* relative to the pelvis. ***r***_*CoM*_ is the vector from the origin to the CoM. In the case of PV_lab_, the PV matches this vector:

(2) $$\begin{array}{r} r_{PV\text{\_}lab}=r_{CoM} \end{array}$$

Since ***r***_*CoM*_ is defined in 3D, PV_lab_ has 3 degrees of freedom (three coordinates). In the case of PV_foot_, the vector from the origin to the pelvis (***r***_*Pelvis*_) is added and the one to the right foot (***r***_*RFoot*_) is subtracted:

(3) $$\begin{array}{r} r_{PV\text{\_}foot}=r_{CoM}+\;r_{Pelvis}-\;r_{RFoot}=\\ \;\frac{1}{\sum_{i=1}^{N}V_{i}}*\overset{N}{\underset{i=1}{\sum }}r_{i}V_{i}+\;r_{Pelvis}-\;r_{RFoot} \end{array}$$

Since ***r***_*CoM*_, ***r***_*Pelvis*_, and ***r***_*RFoot*_ are defined in 3D, PV_foot_ has 3 degrees of freedom (three coordinates).

#### Decomposition of Variability in T, N, and C

Within the TNC approach, changes in PV variability are assigned to changes in one of three components: Tolerance, Noise or Covariation. Tolerance (T) involves changes in the mean configuration of the EV's so it could be seen as a measure for sensitivity; Noise (N) involves changes in the dispersion of the EV's, so how changes in the scattering of the EV's influence the PV; Covariation (C) involves changes in compensatory mechanisms among the EV's so whether the EV's co-vary in a manner that variability of the PV is diminished (or not) (Müller and Sternad, 2004). A TNC analysis is performed at one discrete point in time. Thus, we time-normalized our stance phases and assumed, that over several repetitions, the same posture is specified at a specific percentage of the gait cycle (Scholz and Schöner, 1999). A separate TNC analysis was performed at each time point of the time-normalized stance phase. Afterwards, the means for the absorption and propulsion phase were calculated.

Using the TNC approach, changes in variability of the PV between the two states can be separated into changes due to T, N, and C. To calculate the contributions of these components, five datasets (D1 – D5) are needed (Müller and Sternad, 2004). The CV as a measure of variability is determined for each of the datasets. By comparing the variability calculated with the different datasets, one can attribute changes in PV-variability to one of the three components. All of these datasets consist of the values for our EV's for all subjects, all cycles and all time points:

-D1: measured EV's in the first (PRE) state.
-D2: Data from D1 but permuted over repetitions so that all possible covariance is eliminated (Müller and Sternad, 2003). These data are on the position of D1 in the EV space and have the same dispersion as D1 but no covariation. We used 1,000 permutations.
-D3: Data from D2 but moved to the position of D5 in the EV space (the mean values from D1 are subtracted and the mean values from D5 are added). These data are on the position of D5 in the EV space but with the dispersion of D1 and without covariation.
-D4: Data from D5 but permuted over repetitions so that all possible covariance is eliminated. These data are on the position in the EV space of D5 and have the same dispersion as D5 but without covariation.
-D5: measured EV's in the second (POST) state.

For each of these five datasets the CV as a measure of variability is calculated using our forward model [see Equations (1)–(3)]. When comparing the errors obtained with the five datasets, changes in variability of the PV from the PRE state to the POST state can be analyzed with respect to T, N, and C. By comparing the PV-variability for D1 with the PV-variability for D5, one can see if the variability of the PV changed between the PRE and the POST state. However, one cannot yet state by which component (T, N, or C) this change is caused. It would even be possible that we have changes in the components without observing them on the PV level, because one component causes an increase and another a decrease in PV variability. To determine the changes due to T one has to subtract the PV-variability for D2 from the PV-variability for D3. Since the two data sets have the same dispersion and no covariation, the mean value of the EV's (thus the position in EV space) is the only difference. If changes due to T are observed, the positions in EV space between the PRE and the POST state show difference in error-tolerance so in sensitivity. To determine whether the scattering of the EV cause changes in variability of the PV, the PV-variability for D3 is subtracted from the PV-variability for D4. Since D3 and D4 have no covariation and the same mean value (both are on the POST-position in EV space), the scattering of the EV's is the only difference. So, if changes due to N are observed, the scattering of the EV's between the PRE and the POST state leads to changes in PV-variability. To calculate changes due to changes in covariation among EV's due to fatigue, one has to calculate the differences in PV-variability between D1 and D2 as well as D5 and D4. The only difference between D1 and D2 as well as D5 and D4 is that the data in D4 and D2 were randomized to delete all covariation. So, if changes due to C are observed, changes in covariation among the EV's lead to changes in PV-variability. A positive value for a component signifies that variability increased from state one to state two due to this factor, a negative value that it decreased.

### Statistics

The independent variable is fatigue (PRE vs. POST). The dependent variables are the 3D-length of PV_lab_ and PV_foot_ and the lengths of the projections in the three coordinate axes. Further dependent variables are the CV's of these lengths and the components T, N, C (in %). We calculated a mean value for each dependent variable for the absorption and propulsion phase separately. For the lengths and their CV's we calculated dependent *t*-tests (between PRE and POST). For T, N, and C we calculated one-sample *t*-tests to detect deviations from zero, since these values are a measure for the changes from PRE to POST. Cohen's d was used to indicate effect size for the *t*-tests. A small effect size was d <0.5, a medium effect size was between 0.5 and 0.8 and a large effect size was d > 0.8 (Cohen, 1992). *P* < 0.05 were considered statistically significant.

## Results

The results are shown separately for the two PV's. First, we show the results for PV_lab_ (CoM relative to the lab coordinate system), then we show the results for PV_foot_ (CoM relative to the right foot).

### PV_Lab_

**Figure 2:**
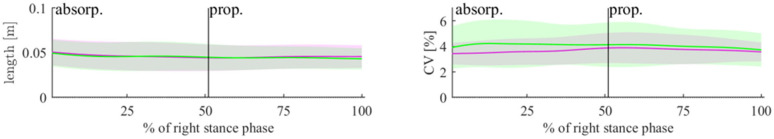
Length of the 3D-vector for PVlab in the PRE (magenta) and POST (green) state (left plot) and the CV of this length (right plot). The lines represent means and the shaded areas represent standard deviations.

Concerning PV_lab_ and its CV, there were no significant effects of fatigue in 3D (see Figure 2 and Table 1). Concerning T, N, and C, only component N showed significant effects of fatigue. An increase in variability due to N with a medium effect size was seen in the absorption phase, although not reaching statistical significance (*p* = 0.096, *d* = 0.501).

**Table 1:** Variability of the dependent variables for PV_lab_ are shown here for PRE and POST (mean ± standard deviation).

		**Absorption**				**Propulsion**			
		**Non-fatigued**	**Fatigued**	***p***	***d***	**Non-fatigued**	**Fatigued**	***p***	***d***
3D	Length [m]	0.047 ± 0.015	0.046 ± 0.015	0.748	0.091	0.045 ± 0.013	0.044 ± 0.012	0.459	0.212
	CV [%]	3.606 ± 0.909	4.147 ± 1.587	0.215	0.363	3.765 ± 0.994	4.000 ± 1.402	0.584	0.156
	T [%]	0.003 ± 0.014		0.553	0.169	0.004 ± 0.014		0.388	0.249
	N [%]	0.013 ± 0.025		0.096	**0.501**	0.007 ± 0.033		0.471	0.206
	C [%]	0.525 ± 1.411		0.222	0.357	0.224 ± 1.417		0.594	0.152
Anterior-posterior	Length [m]	0.033 ± 0.015	0.033 ± 0.015	0.673	0.120	0.035 ± 0.013	0.035 ±0.013	0.987	0.004
	CV [%]	4.668 ± 0.886	5.091 ± 1.970	0.368	0.259	4.289 ± 0.969	4.666 ± 1.555	0.358	0.265
	T [%]	0.002 ± 0.018		0.695	0.111	0.001 ± 0.014		0.753	0.089
	N [%]	0.008 ± 0.029		0.391	0.247	0.011 ± 0.030		0.234	0.347
	C [%]	0.413 ± 1.546		0.373	0.257	0.365 ± 1.339		0.363	0.262
Medio-lateral	Length [m]	0.022 ± 0.010	0.022 ± 0.009	0.796	0.073	0.010 ± 0.004	0.011 ± 0.005	0.131	0.450
	CV [%]	4.563 ± 1.145	4.796 ± 1.347	0.396	0.244	4.296 ± 0.712	5.292 ± 1.242	**0.012**	**0.822**
	T [%]	0.003 ± 0.013		0.474	0.205	0.001 ± 0.012		0.736	0.096
	N [%]	0.013 ± 0.021		0.051	**0.602**	0.025 ± 0.040		0.051	**0.602**
	C [%]	0.218 ± 0.896		0.417	0.233	0.970 ± 1.135		**0.012**	**0.821**
Vertical	Length [m]	0.019 ± 0.011	0.018 ± 0.010	0.179	0.395	0.023 ± 0.013	0.021 ± 0.011	0.092	**0.507**
	CV [%]	2.696 ± 0.462	3.143 ± 1.288	0.195	0.381	2.872 ± 0.755	3.257 ± 1.668	0.322	0.287
	T [%]	0.004 ± 0.009		0.130	0.450	0.005 ± 0.011		0.129	0.452
	N [%]	0.014 ± 0.026		0.095	**0.502**	0.011 ± 0.030		0.219	0.360
	C [%]	0.430 ± 1.104		0.203	0.374	0.369 ± 1.264		0.332	0.280

*Moderate or strong effect sizes and significant p-values are highlighted in bold. There is only one value for T, N, and C, since they describe the change from PRE to POST. A negative value signifies a decrease in variability, positive values an increase. CV represents the coefficient of variation and T, N, C, the components tolerance, noise, and covariation*.

In the anterior-posterior direction, there were no significant effects of fatigue.

In the medio-lateral direction, the CV increased with fatigue in the propulsion phase (*p* = 0.012, *d* = 0.822). Component N showed an increase in variability with a medium effect size in both phases, although not reaching statistical significance (both *p* = 0.051, *d* = 0.602). Component C showed a significant increase in variability during propulsion phase with a high effect size (*p* = 0.012, *d* = 0.821).

In the vertical direction, PV_lab_ decreased with fatigue with a medium effect size during propulsion phase, although not reaching statistical significance (*p* = 0.092, *d* = 0.507). There were no significant effects on the CV of PV_lab_. Only factor N showed an increase with a medium effect size during absorption, although not reaching statistical significance (*p* = 0.095, *d* = 0.502).

Summarizing these results, the only significant effects of fatigue on PV_lab_ are a decrease in vertical direction which does not reach statistical significance. Hypothesis (1) can thus be accepted.

### PV_Foot_

**Figure 3:**
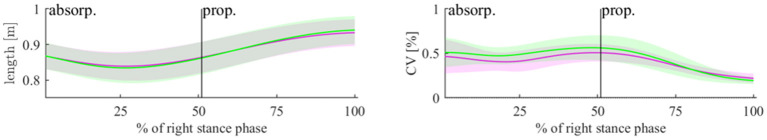
Length of the 3D-vector for PVfoot in the PRE (magenta) and POST (green) state (left plot) and the CV of this length (right plot). The lines represent means and the shaded areas represent standard deviations.

Concerning PV_foot_, there was a significant decrease in the absorption phase (*p* = 0.035, *d* = 0.658) and a significant increase in the propulsion phase (*p* = 0.045, *d* = 0.621), both with a medium effect size. The CV of PV_foot_ increased during the absorption phase (*p* = 0.027, *d* = 0.696) with a medium effect size (see Figure 3 and Table 2). Concerning T, N, and C, only component T showed significant effects of fatigue. A decrease in variability due to T was seen in the absorption phase (*p* < 0.001, *d* = 1.488) and in the propulsion phase (*p* = 0.028, *d* = 0.693). The component N showed an increase in variability during absorption with a medium effect size without reaching statistical significance (*p* = 0.057, *d* = 0.583).

**Table 2:** The values of the dependent variables for PV_foot_ are shown here for PRE and POST and for absorption and propulsion (mean ± standard deviation).

		**Absorption**				**Propulsion**			
		**PRE**	**POST**	***p***	***d***	**PRE**	**POST**	***p***	***d***
3D	Length [m]	0.849 ± 0.038	0.846 ± 0.040	**0.035**	**0.658**	0.905 ± 0.039	0.908 ± 0.040	**0.045**	**0.621**
	CV [%]	0.451 ± 0.105	0.514 ± 0.109	**0.027**	**0.696**	0.363 ± 0.055	0.376 ± 0.096	0.618	0.142
	T [%]	−0.003 ± 0.002		** <0.001**	**1.488**	−0.001 ± 0.002		**0.028**	**0.693**
	N [%]	0.058 ± 0.096		0.057	**0.583**	0.026 ± 0.065		0.190	0.386
	C [%]	0.007 ± 0.061		0.688	0.114	−0.012 ± 0.062		0.517	0.185
Anterior-posterior	Length [m]	0.116 ± 0.010	0.118 ± 0.010	0.148	0.429	0.427 ± 0.031	0.440 ± 0.031	** <0.001**	**1.621**
	CV [%]	1.672 ± 0.673	1.476 ± 0.491	0.120	0.464	1.023 ± 0.234	0.959 ± 0.320	0.597	0.150
	T [%]	−0.004 ± 0.003		** <0.001**	**1.285**	−0.004 ± 0.003		** <0.001**	**1.236**
	N [%]	−0.193 ± 0.452		0.165	0.410	−0.034 ± 0.375		0.761	0.086
	C [%]	−0.027 ± 0.068		0.196	0.379	−0.026 ± 0.098		0.382	0.252
Medio-lateral	Length [m]	0.015 ±0.010	0.013 ± 0.009	0.599	0.150	0.015 ± 0.008	0.016 ± 0.011	0.684	0.116
	CV [%]	0.891 ± 0.222	0.851 ± 0.294	0.574	0.160	0.826 ± 0.179	0.794 ± 0.149	0.575	0.160
	T [%]	0.013 ± 0.044		0.314	0.292	0.043 ± 0.183		0.517	0.185
	N [%]	−0.036 ± 0.237		0.608	0.146	0.015 ± 0.229		0.823	0.064
	C [%]	−0.017 ± 0.067		0.403	0.241	−0.040 ± 0.067		0.061	**0.574**
Vertical	Length [m]	0.838 ± 0.037	0.834 ± 0.039	**0.041**	**0.634**	0.793 ± 0.035	0.789 ± 0.037	**0.009**	**0.865**
	CV [%]	0.376 ± 0.103	0.474 ± 0.094	**0.004**	**0.994**	0.459 ± 0.099	0.535 ± 0.154	0.095	**0.503**
	T [%]	−0.002 ± 0.223		**0.002**	**1.127**	−0.002 ± 0.001		**0.001**	**1.152**
	N [%]	0.093 ± 0.104		**0.009**	**0.861**	0.065 ± 0.141		0.139	0.440
	C [%]	0.006 ± 0.062		0.734	0.096	0.013 ± 0.047		0.356	0.266

*Moderate or strong effect sizes and significant p-values are highlighted in bold. There is only one value for T, N, and C, since they describe the changes from PRE to POST. A negative value signifies a decrease in variability, positive values an increase. CV represents the coefficient of variation and T, N, C the components tolerance, noise, and covariation*.

In the anterior-posterior direction, there was an increase in PV_foot_ in the propulsion phase (*p* < 0.001, *d* = 1.621). The CV of PV_foot_ was not affected by fatigue. Component T showed a decrease in variability during the absorption phase (*p* < 0.001, *d* = 1.285) and propulsion phase (*p* < 0.001, *d* = 1.236).

In the medio-lateral direction, there were no significant effects of fatigue on PV_foot_ or its CV. Component C showed a decrease in variability during propulsion with a medium effect size but without reaching statistical significance (*p* = 0.061, *d* = 0.574).

In the vertical direction, PV_foot_ decreased during both absorption (*p* = 0.041, *d* = 0.634) and propulsion (*p* = 0.009, *d* = 0.865). The CV increased during absorption phase (*p* = 0.004, *d* = 0.994). In the propulsion phase, there was also an increase with a medium effect size but without reaching statistical significance (*p* = 0.095, *d* = 0.503). Significant changes were observed in components T in both phases (abs.: *p* = 0.002, *d* = 1.127; prop.: *p* = 0.001, *d* = 1.152) and N during absorption phase (*p* = 0.009, *d* = 0.861).

Since PV_foot_ was affected by fatigue in 3D and in anterior-posterior and in vertical direction, hypothesis (2) could be accepted.

## Discussion

The purpose of this study was to investigate if and how runners adjust their coordination as reaction to fatigue when running at constant speed and how this fatigue affects the variability of the CoM. Additionally, we wanted to compare the results of the TNC analysis with results obtained with the UCM approach in an earlier study (Möhler et al., 2019). Therefore, we performed a TNC analysis with two different PV's: PV_lab_ is the global CoM relative to the origin. This PV was chosen to be able to compare our results to the ones obtained with the UCM. PV_foot_ is the CoM relative to the right foot. This PV was chosen since we think that is functionally more relevant, since it describes the relation between the foot and the CoM which is crucial for the forward propulsion during running.

To be able to combine the position of the foot and the joint angles in our analysis, we chose the TNC approach, since this approach is performed in the results space and allows for the combination of EV's of different units. Our hypotheses were confirmed, since we found no effects of fatigue for PV_lab_ in 3D, but there were effects of fatigue for PV_foot_ both in 3D and in the projections.

In the following, we will discuss the findings of the TNC analysis for PV_lab_ and PV_foot_ and then comment on some methodological consideration concerning the comparison between the UCM and the TNC approach. Afterwards, we will address the limitations of our study and comment on its contributions to the field.

### Fatigue Effects on CoM Trajectory and Its Variability

We analyzed the effects of fatigue on the CoM trajectory and its variability using two different PV's: PV_lab_, where the position of the CoM is described relative to a lab coordinate system and PV_foot_, where the CoM is described relative to the right foot.

Concerning PV_lab_, the only changes with fatigue visible in 3D were a non-significant increase in variability due to the component N with a medium effects size. There were no effects in anterior-posterior direction. In the medio-lateral direction, the stride-to-stride variability of the CoM trajectory increased during propulsion phase, due to increases in variability caused by the components N and C. In the POST state, the CoM was lower during propulsion phase than during the PRE state. These results show that relative to a fixed point, runners lowered their CoM slightly in the POST state and showed more stride-to-stride variability in the medio-lateral direction due to a less error-tolerant joint configuration and more variability in the joint angles.

Concerning PV_foot_, the results show that in 3D, the distance between the right foot and the CoM decreased during absorption and increased during propulsion phase. The decrease in distance can be explained by a lower CoM (decrease in vertical direction). The increase was due to an increase in anterior-posterior direction. The stride-to-stride variability of the CoM trajectory increased during absorption phase, caused by more variability in the vertical direction, which was caused by an increase in component N. This means that changes in the dispersion of the joint angles caused this increase. Component T caused a decrease in variability in both absorption and propulsion phase in the anterior-posterior and vertical direction as well as in 3D. This means that runners had a less error-tolerant joint configuration, especially in the sagittal plane. The effects of this component however were considerably smaller than the ones of N and C, so the effects of T might have been hidden and are thus not visible as a decreased CoM variability.

At first sight, the changes in CoM trajectory in this study are contradicting the results of Girard et al. (2013), who found that runners kept their CoM on the same height. However, it must be noted that runners were able to adapt their running speed in the study by Girard et al. (2013), which they could not in the present study. So apparently, runners choose a different strategy when running at a constant speed. Stride-to-stride variability of the CoM trajectory increased with fatigue. A high variability can indicate changes in running style, which potentially increase energy consumption caused by deviation from the individual's optimal running style (Williams and Cavanagh, 1987; Moore, 2016). Thus, runners were probably less economical in the POST state. The changes in the vertical direction might be explained by reductions in leg stiffness which are commonly observed in a POST state (Dutto and Smith, 2002; Rabita et al., 2011, 2013; Girard et al., 2013). Reductions in leg stiffness have also been linked to a lower running economy (Dalleau et al., 1998). A decreased stiffness could also explain the reduced distance from the right foot to the CoM during absorption phase, since runners would have a more compliant leg at touchdown, so the CoM was lowered (decreases in length in the vertical direction). Since the speed was fixed, runners had to push longer in order to keep up, which explains the increased distance in the propulsion phase visible in the anterior-posterior direction.

We find more changes with fatigue when analyzing PV_foot_ than when analyzing PV_lab_, so changes with fatigue are more pronounced in the CoM trajectory relative to the right foot than in the CoM trajectory relative to a fixed point. This might be either caused by changes in foot position or in the position of the pelvis or both. Hoenig et al. (2019) found increases in local dynamic stability of the pelvis with fatigue, so one might assume that it was especially the foot motion which changed with fatigue. In the analysis using PV_lab_, an increased variability in medio-lateral direction was detected which is not visible when analyzing PV_foot_. So, it is only visible with respect to a fixed reference. This could mean that runners move medio-laterally on the treadmill. Since we focussed on stride-to-stride variability, we cannot comment on effects of fatigue on the vertical oscillation or the medio-lateral movement of the CoM throughout the gait cycle.

### Methodological Considerations—References to the UCM Results

The TNC analysis with PV_lab_ as PV was performed to be able to compare the results obtained here with those obtained using an UCM analysis (Möhler et al., 2019). The results corroborated those obtained with the UCM analysis, as we found no effects on the PV_lab_ in 3D. The only effect we found for T, N, or C was an increase in variability with a medium effect size due to N in the absorption phase which did not reach statistical significance (*p* = 0.096, *d* = 0.501). The analysis of stride-to-stride variability with respect to PV_foot_ would not be possible within the UCM, since we would have to combine the different units of the foot and pelvis location in meters with the joint angles in degrees or radians. This is not feasible within the UCM approach (Latash et al., 2007). When calculating the Jacobian, which is the core of the UCM analysis, the position of the foot or pelvis would disappear when performing the partial derivatives since they are not expressed in dependence on any EVs (e.g., a constant term without any dependency). The CoM trajectory within the UCM approach is not suitable to describe its movement along its trajectory in a global coordinate system because it is rather a parameter representing fluctuation of the CoM around an arbitrary point in the coordinate system. Therefore, one should calculate the CoM trajectory of interest separately when a UCM analysis is performed.

With the TNC approach used in this study, the combination of different units within the EV's does not pose a problem since the analysis is performed in the result space (Müller and Sternad, 2009). Although the possibility to analyze variability on the level of the whole human body is a big strength of these approaches, analyzing sub-systems can also lead to deeper insights. Within the TNC approach, the analysis of the projections in the three dimensions is possible since it is performed in the result space (Müller and Sternad, 2009). Within the UCM approach a separate model for each dimension would have to be built up.

There are some other differences between the two approaches. When performing an UCM analysis one should select a set of EV's that show no task-independent covariation (Latash et al., 2007). Covariance inherent to the system will be detected by the UCM as parallel variance, although it might only be an artifact of the musculoskeletal system and might not arise from motor control processes. To determine the Jacobian necessary for an UCM analysis, the forward model has to be linearized. This means that only differentiable forward models can be implemented (Müller and Sternad, 2009). Whether this linearization is feasible could be studied by comparing the results of the linearized model with the full forward model (Scholz and Schöner, 1999). However, the influence of the linearization is rarely examined. The orthogonal variance is determined based on this linearization (Latash et al., 2007), but orthogonality is only given in a Euclidean space and it is hard to determine whether this assumption is valid. Performing an UCM analysis without having examined whether these requirements are met does not mean that the analysis will lead to wrong or unusable results, although the influence of violating these assumptions is hard to evaluate. While an UCM analysis tests a hypothesis concerning the degree of control or stability of a certain PV (Latash et al., 2007), a TNC analysis only quantifies the influence of components T, N, and C on the variability of the result. Hypotheses about control have to be subsequently analyzed. Also, variability not affecting the PV (in the UCM context: parallel variability) is not detected within the TNC approach, since it is not captured by T, N, or C (Schöner and Scholz, 2007). Therefore, the two approaches are not in conflict, but are instead complementary since both look at a given problem from different perspectives. The UCM analysis can be performed on data from a single measurement. The TNC analysis was developed in the context of motor learning and always shows a development from one state to another, so it cannot be performed on single measurements.

Some parallels can be drawn between the results obtained with the two approaches. Changes in component N in a TNC analysis can be seen in an UCM analysis as changes in the orthogonal variance. Changes in component C could be seen as changes in the repartition of variance on the parallel and orthogonal components and so in the UCM ratio. Verrel (2011) showed that, in 1D, the measure for covariation is even equivalent between the two approaches. Changes in component T are not detectable in the UCM since the forward model is linearized around the mean configuration, which has no effect on UCM results.

### Limitations

To capture consecutive gait cycles, this study was performed on a treadmill. There are a number of studies showing differences between treadmill running and overground running (Fellin et al., 2012). Given the time of treadmill familiarization (Matsas et al., 2000; Lavcanska et al., 2005), it can be assumed that movement patterns were at least stable and differences from overground running were minimal (Riley et al., 2008). However, the constant speed of the treadmill is expected to result in less variability in the movement execution.

So far, the TNC approach, as well as the UCM approach have been mainly used to analyze postures at one specific moment in time. Here we apply these approach to a whole-body continuous movement (comparable to Hamacher et al., 2019; Yamagata et al., 2019). In order to do so, we had to time-normalize our data, although we acknowledge the fact that this might mask certain variability in timing over the stance phase.

Even if we find statistically significant and thus systematic effect with fatigue on the CoM trajectory, one has to critically question the practical significance of the findings. The observed effects in this study have to be considered as small (differences in distance of ± 3 mm, differences in CV <1%). However, since we study trained runners we cannot expect huge changes. Also, since runners had to run at a high, fixed speed (19.27 ± 0.72 km/h), maintaining the speed has not allowed any major deviations. Due to the limitation to male runners and the relatively small sample size, our findings are not directly generalizable or transferable to other samples such as recreational athletes or female runners.

Since the TNC and other related variability analyses are always coordinate dependent (Schöner and Scholz, 2007; Sternad et al., 2010), we have to emphasize that our results are only valid for the chosen coordinates. We analyzed the trajectory of the CoM with respect to two different coordinate systems during the stance phase and this analysis was performed in the coordinate space spanned by our EV's—the joint angles. The results for an analysis performed in a different coordinate frame might differ. We chose the joint angles as EV's since they are a possible control variable during motor control, in agreement with other studies (Papi et al., 2015; Hamacher et al., 2019; Yamagata et al., 2019).

### Conclusion and Outlook

For the first time, the TNC analysis was used in the context of running as well as in combination with an 3D full body model. The results obtained with this approach were compared with results obtained with the UCM approach on the same dataset and their differences and similarities were outlined.

Concerning PV_lab_ we found that runners increased their stride-to-stride variability in medio-lateral direction by 1%. Looking at PV_foot_ we found that runners lowered their CoM by 4 mm and increased their stride-to-stride variability in the absorption phase in both 3D and in the vertical direction. The lowering of the CoM might be explained by a reduced leg stiffness. Apparently, runners have to lower their CoM in order to maintain a fixed running speed throughout a fatiguing run.

Both the UCM and the TNC approach were developed and are mainly applied in well-controlled lab movements with limited degrees of freedom, in contrast to our application to a complex whole body movement. Even though this is a necessary, results from these experiments are not always transferable to whole-body sports movements. In this study, we show that this transfer is feasible. Even though we only find minor effects in our study, these methods are promising approaches to gain further insights into the stride-to-stride variability in running.

## Data Availability Statement

The data analyzed in this study is subject to the following licenses/restrictions: the raw data supporting the conclusions of this article will be made available by the authors, without undue reservation. Requests to access these datasets should be directed to felix.moehler@kit.edu.

## Ethics Statement

The studies involving human participants were reviewed and approved by Ethics Committee of the Karlsruhe Institute of Technology. The patients/participants provided their written informed consent to participate in this study.

## Author Contributions

FM: conceptualization, methodology, software, formal analysis, investigation, data curation, writing-original draft, writing-review and editing, visualization, and project administration. BS and HM: conceptualization, methodology, and writing-review and editing. TS: conceptualization, methodology, resources, writing-review and editing, supervision, and funding acquisition. All authors contributed to the article and approved the submitted version.

## Conflict of Interest

The authors declare that the research was conducted in the absence of any commercial or financial relationships that could be construed as a potential conflict of interest.
